# Understanding Gait, Its Neurophysiologic Control and Systematic Localisation of Gait Abnormalities

**DOI:** 10.21315/mjms-05-2025-406

**Published:** 2025-12-31

**Authors:** Thaanesh Manokaran, Muhammad Zikri Yusoff, Prehmanraj Mariyapan, Jafri Malin Abdullah

**Affiliations:** 1 Department of Neurosciences, School of Medical Sciences, Universiti Sains Malaysia, Health Campus, Kelantan, Malaysia; 2 Brain Behaviour Cluster, Universiti Sains Malaysia Specialist Hospital and School of Medical Sciences, Universiti Sains Malaysia, Health Campus, Kelantan, Malaysia

**Keywords:** gait, central nervous system, abnormal gait, gait cycle, gait disorder, neural control

## Abstract

Gait analysis is a crucial diagnostic tool in neurology and rehabilitation, reflecting the intricate coordination between the central and peripheral nervous systems, musculoskeletal structures, and sensory feedback mechanisms. This article integrates the neurophysiological basis of gait, emphasising the role of central pattern generators in the spinal cord and the mesencephalic locomotor region in regulating initiation, speed, and adaptability (1). Gait disorders result from disruptions in various neural structures, including the cerebellum, basal ganglia, motor and sensory pathways, and the peripheral nervous system (2). Functional gait disorders, characterised by inconsistent or exaggerated patterns, require differentiation from organic conditions (3). This article also highlights diagnostic tools, including motion capture systems, electromyography, and wearable sensors, as well as therapeutic interventions, such as physical therapy, pharmacological treatments, and emerging rehabilitative technologies (4).

## Introduction

Human gait is a complex, coordinated motor activity that integrates multiple neural structures, including the cerebellum, basal ganglia, brainstem, spinal cord, and peripheral nerves (5). Disruptions in these systems result in pathological gait patterns, which provide essential clues for diagnosing neurological or musculoskeletal disorders (6). The gait cycle, divided into stance (60%) and swing (40%) phases, relies on rhythmic motor patterns generated by central pattern generators and modulated by descending supraspinal inputs (7).

## Neurophysiology of Gait

### Normal Gait Cycle

The human gait cycle consists of two main phases – stance and swing. Each phase is further divided into specific subphases that are essential for efficient locomotion (2).

#### Stance Phase (60% of Gait Cycle)

- Initial contact: The heel strikes the ground, stabilising the limb (6).
- Loading response: Weight transfers onto the limb, requiring knee flexion control by the quadriceps (4).
- Midstance: The body advances over the supporting limb, with the gastrocnemius and soleus stabilising the tibia.
- Terminal stance: The heel rises off the ground, propelling forward with assistance from the plantar flexors.
- Pre-swing: The limb prepares for the swing phase using the rectus femoris and iliopsoas for hip flexion.

#### Swing Phase (40% of Gait Cycle)

- Initial swing: The foot lifts off the ground, clearing obstacles via dorsiflexion (1).
- Midswing: The thigh advances forward with continued knee and hip flexion.
- Terminal swing: The hamstrings decelerate limb movement, preparing for initial contact again.

**Figure 1:**
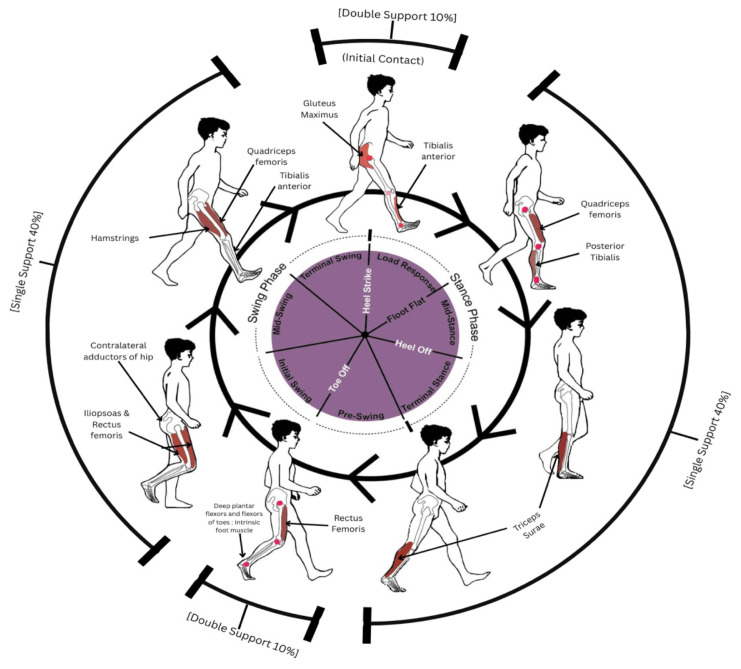
Phases of gait cycle and key muscle activation

Proper coordination between these phases ensures smooth locomotion, relying on neural input from the cerebellum, basal ganglia, and sensory pathways (5). Figure 1 illustrates the gait cycle divided into stance (60%) and swing (40%) phases. Key lower limb muscles active during each subphase are highlighted, including the gluteus maximus, quadriceps, hamstrings, tibialis anterior, triceps surae, and hip flexors. Arcs indicate periods of double- and single-limb support.

### Normal Gait Cycle Requirements

#### Central Pattern Generators (CPGs)

Located in the spinal cord, these neural networks produce rhythmic locomotor patterns essential for walking (4).

#### Mesencephalic Locomotor Region (MLR)

Regulates gait initiation and speed via reticulospinal pathways (1).

#### Basal Ganglia and Cerebellum

Modulate movement coordination, amplitude, and postural stability (5).

#### Sensory Feedback Systems

Proprioceptive, visual, and vestibular inputs ensure real-time adjustments for balance and environmental adaptation (2).

Key neural structures involved in gait control:

- Higher centres (PFC, SMA, M1) initiate and plan movement.
- Basal ganglia modulate automaticity and selection.
- The cerebellum ensures coordination and adaptation.
- MLR initiates rhythmic patterns via the spinal CPGs, which generate and coordinate locomotor output.

### Pathophysiology of Gait Abnormalities and Localisation

Gait abnormalities arise from dysfunction in neural pathways, leading to characteristic movement patterns that provide critical diagnostic insights. Understanding these disruptions enhances the ability to localise the underlying pathology.

### Cerebellar Gait Disorders – Ataxic Gait

Caused by lesions in the cerebellum, particularly the spinocerebellum and vestibulocerebellum, this gait is wide-based and unsteady, with irregular step timing (7). Spinocerebellar damage results in dysmetria and impaired limb coordination, while vestibulocerebellar lesions cause significant truncal instability and swaying (8).[Fig f2-15mjms3206_sc]

#### Basal Ganglia Dysfunction and Parkinsonian Gait

- Parkinsonian gait: Due to substantia nigra degeneration, this gait is characterised by rigidity, shuffling steps, festination, and freezing episodes, indicative of basal ganglia dysfunction (9). Loss of dopaminergic modulation leads to decreased stride length and difficulty initiating movement.
- Hyperkinetic gaits: Disorders such as Huntington’s disease and dystonia lead to athetotic, choreic, and dystonic gait patterns, manifesting as involuntary excessive movements disrupting normal walking (10).

#### Motor and Sensory Tract Lesions

- Spastic gait: Results from corticospinal tract damage, such as in multiple sclerosis or stroke, causing hypertonia and scissoring of the legs (11). Impaired voluntary control results in stiff, jerky movements with limited knee flexion.
- Hemiparetic gait: Typical in stroke patients, this gait involves unilateral weakness, circumduction of the affected limb, and reduced arm swing, indicative of upper motor neuron damage (12).
- Sensory ataxia: Due to dorsal column dysfunction, patients rely on visual feedback, leading to a high step gait with heavy heel strikes. A positive Romberg sign confirms proprioceptive deficits (13).
- Vestibular ataxia: Vestibular system damage causes veering gait, worsened by eye closure and characterised by imbalance and falling towards the lesion side (14).

#### Peripheral Neuropathies and Musculoskeletal Gaits

- Steppage gait: Associated with peroneal nerve palsy or severe neuropathy, causing foot drop. Patients lift the affected leg high to prevent dragging (5).
- Waddling gait: Seen in muscular dystrophies, characterised by excessive trunk movement and difficulty stabilising the pelvis due to proximal muscle weakness (15).

#### Functional Gait Disorders

These disorders present inconsistent patterns and exaggerated movements without a clear neurological basis. Often psychogenic in origin, they include sudden knee buckling, hesitation, and improved gait when distracted (5).

**Figure 3:**
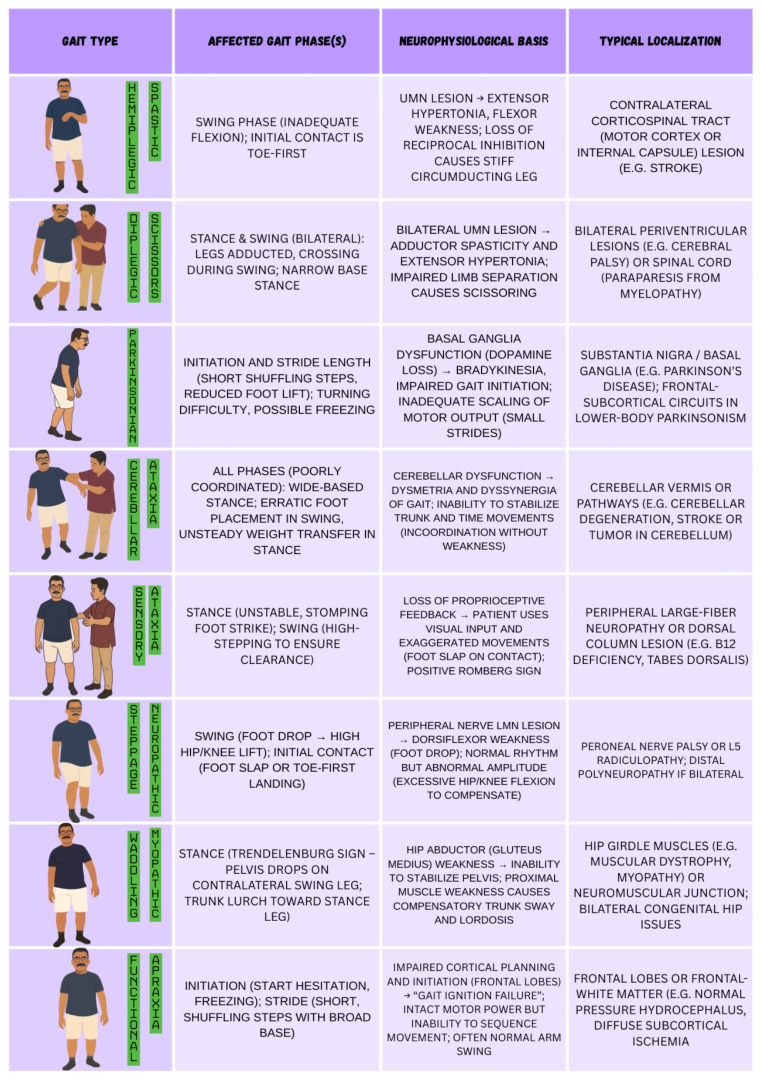
Classification of abnormal gait types by phase, neurophysiology, and localisation

Figure 3 summarises common pathological gait types, detailing the affected gait phases, underlying neurophysiological mechanisms, and typical anatomical localisations. Gait patterns include hemiplegic, scissoring, Parkinsonian, cerebellar and sensory ataxia, steppage, waddling, apraxic, and functional gait, providing a structured framework for clinical localisation.

## Management Strategies

### Pharmacological Therapy

Dopaminergic medications for Parkinsonian gait and muscle relaxants for spasticity (16).

### Physical Therapy and Rehabilitation

Strength, balance, and coordination training (17).

### Assistive Devices

Canes, walkers, and orthotics to aid mobility (18).

### Emerging Technologies

Robotic-assisted gait training and neuromodulation to improve rehabilitation outcomes (1).

As a supplement to this article, we provide a link to a video of gait examination and several common forms of gait pathology encountered in our practice (duration 18 minutes).

Available at YouTube: https://www.youtube.com/watch?v=jv8lfuAIVhU

## Conclusion

A structured understanding of gait pathophysiology aids in diagnosing and managing movement disorders. Advances in diagnostic tools and therapies enhance patient outcomes by providing targeted interventions. A multidisciplinary approach that incorporates neurology, physiotherapy, and rehabilitative technologies optimises functional mobility and quality of life.

## Figures and Tables

**Figure 2 f2-15mjms3206_sc:**
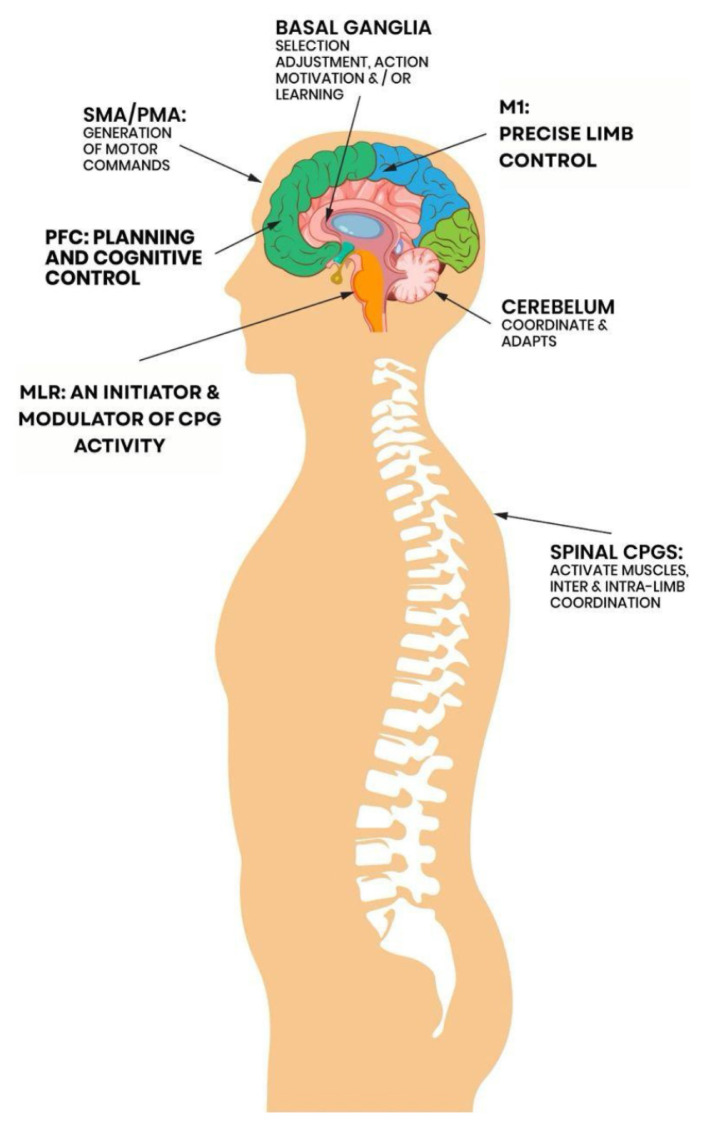
Neural control of gait

## References

[1] Takakusaki K (2013). Neurophysiology of gait: from the spinal cord to the frontal lobe. Mov Disord.

[2] Perry J, Burnfield JM (2010). Gait analysis: normal and pathological function.

[3] Nonnekes J, Ruzicka E, Serranova T, Reich SG, Bloem BR, Hallett M (2020). Functional gait disorders: A sign-based approach. Neurology.

[4] Minassian K, Hofstoetter US, Dzeladini F, Guertin PA, Ijspeert A (2017). The human central pattern generator for locomotion: does it exist and contribute to walking?. Neuroscientist.

[5] Schweizer K (2013). Principles of pathological gait [dissertation].

[6] Kuo AD, Donelan JM (2010). Dynamic principles of gait and their clinical implications. Phys Ther.

[7] Takakusaki K, Takahashi M, Obara K, Chiba R (2017). Neural substrates involved in the control of posture. Adv Robot.

[8] Gilman S (1992). The cerebellum and motor dysfunction. J Neurol Neurosurg Psychiatry.

[9] Jankovic J, Nutt JG, Sudarsky L (2001). Classification, diagnosis, and etiology of gait disorders. Adv Neurol.

[10] Bhatia KP, Marsden CD (1994). The behavioural and motor consequences of focal lesions of the basal ganglia in man. Brain.

[11] Lee TT, Manzano GR, Green BA (1997). Modified open-door cervical expansive laminoplasty for spondylotic myelopathy: operative technique, outcome, and predictors for gait improvement. J Neurosurg.

[12] Graff-Radford N, Godersky J, Masdeu J, Sudarsky L, Wolfson L (1997). Gait disorders of aging: falls and therapeutic strategies.

[13] Baloh RW, Yue Q, Socotch TM, Jacobson KM (1995). White matter lesions and disequilibrium in older people. Arch Neurol.

[14] Camicioli R, Moore MM, Sexton G, Howieson DB, Kaye JA (1999). Age-related brain changes associated with motor function in healthy older people. J Am Geriatr Soc.

[15] Victor M, Ferrendelli JA, Fields W, Willis W (1970). The nutritional and metabolic diseases of the cerebellum: Clinical and pathological aspects. The cerebellum in health and disease.

[16] Compta Y, Valldeoriola F, Tolosa E, Rey MJ, Marti MJ, Valls-Sole J (2007). Long lasting pure freezing of gait preceding progressive supranuclear palsy: A clinicopathological study. Mov Disord.

[17] Sudarsky L, Tideiksaar R, Masdeu J, Sudarsky L, Wolfson L (1997). Gait disorders of aging: falls and therapeutic strategies.

[18] Masdeu JC, Masdeu J, Sudarsky L, Wolfson L (1997). Gait disorders of aging Falls and therapeutic strategies.

